# A Molecular Epidemiological Study of *var* Gene Diversity to Characterize the Reservoir of *Plasmodium falciparum* in Humans in Africa

**DOI:** 10.1371/journal.pone.0016629

**Published:** 2011-02-09

**Authors:** Donald S. Chen, Alyssa E. Barry, Aleksandra Leliwa-Sytek, Terry-Ann Smith, Ingrid Peterson, Stuart M. Brown, Florence Migot-Nabias, Philippe Deloron, Moses M. Kortok, Kevin Marsh, Johanna P. Daily, Daouda Ndiaye, Ousmane Sarr, Souleymane Mboup, Karen P. Day

**Affiliations:** 1 Department of Medical Parasitology, New York University School of Medicine, New York, New York, United States of America; 2 Department of Medicine, New York University School of Medicine, New York, New York, United States of America; 3 Peter Medawar Building for Pathogen Research and Department of Zoology, University of Oxford, Oxford, United Kingdom; 4 Centre for Population Health, Burnet Institute, Melbourne, Australia; 5 Department of Medicine, Central and Eastern Clinical School, Monash University, Victoria, Australia; 6 Center for Health Informatics and Bioinformatics, New York University School of Medicine, New York, New York, United States of America; 7 Institut de Recherche pour le Développement, Faculté de Pharmacie, Université Paris 5, Paris, France; 8 Kenya Medical Research Institute, Centre for Geographic Medicine Research, Kilifi, Kenya; 9 Department of Immunology and Infectious Diseases, Harvard School of Public Health, Boston, Massachusetts, United States of America; 10 Department of Medicine, Albert Einstein College of Medicine, Bronx, New York, United States of America; 11 Faculty of Medicine and Pharmacy, Cheikh Anta Diop University, Dakar, Senegal; Agency for Science, Technology and Research (A*STAR), Singapore

## Abstract

**Background:**

The reservoir of *Plasmodium* infection in humans has traditionally been defined by blood slide positivity. This study was designed to characterize the local reservoir of infection in relation to the diverse *var* genes that encode the major surface antigen of *Plasmodium falciparum* blood stages and underlie the parasite's ability to establish chronic infection and transmit from human to mosquito.

**Methodology/Principal Findings:**

We investigated the molecular epidemiology of the *var* multigene family at local sites in Gabon, Senegal and Kenya which differ in parasite prevalence and transmission intensity. 1839 distinct *var* gene types were defined by sequencing DBLα domains in the three sites. Only 76 (4.1%) *var* types were found in more than one population indicating spatial heterogeneity in *var* types across the African continent. The majority of *var* types appeared only once in the population sample. Non-parametric statistical estimators predict in each population at minimum five to seven thousand distinct *var* types. Similar diversity of *var* types was seen in sites with different parasite prevalences.

**Conclusions/Significance:**

*Var* population genomics provides new insights into the epidemiology of *P. falciparum* in Africa where malaria has never been conquered. In particular, we have described the extensive reservoir of infection in local African sites and discovered a unique *var* population structure that can facilitate superinfection through minimal overlap in *var* repertoires among parasite genomes. Our findings show that *var* typing as a molecular surveillance system defines the extent of genetic complexity in the reservoir of infection to complement measures of malaria prevalence. The observed small scale spatial diversity of *var* genes suggests that *var* genetics could greatly inform current malaria mapping approaches and predict complex malaria population dynamics due to the import of *var* types to areas where no widespread pre-existing immunity in the population exists.

## Introduction

Hundreds of millions of *Plasmodium falciparum* infections persist for many months in the human population to sustain malaria transmission where anopheline mosquito vectors are only seasonally available. These chronic, largely undetected, infections constitute the *reservoir* of infection and serve to fuel continued malaria transmission.

During the past era of malaria eradication the size and persistence of the reservoir of infection were considered major obstacles to the eradication of *P. falciparum* in Africa [1]. The duration of a single infection was measured to be 150 to 200 days using models and epidemiological observations of blood smear data [2]. Today, with the advantage of molecular tools, we understand that individuals in endemic areas harbor chronic *P. falciparum* infections composed of several genetically distinct haploid parasite genomes introduced by single and/or multiple (superinfection) mosquito bites [3], [4], [5]. The duration of infection of individual genotypes is influenced by age, prior exposure, coinfection and superinfection [3], [4], [6]. Density-dependent regulation of parasitemia has also been shown to occur among *Plasmodium spp* and genotypes in coinfected semi-immune children [3]. Considering these infection dynamics, the parasite rate (prevalence) as defined by blood slide positivity does not accurately describe the complexity of the reservoir of infection and yet remains the mainstay for monitoring and evaluating malaria control [7]. Consequently, we considered how best to characterize the reservoir of infection in humans using parasite genetics/population genomics.

Diversity measures based on polymorphic antigen encoding genes, barcoding, microsatellite loci and genome-wide single nucleotide polymorphisms [5], [8], [9] define the number of distinct alleles and/or genomes per infected human, the latter termed multiplicity of infection (MOI). They also define the estimated number of distinct genomes in a population. This molecular epidemiological information, while useful to evaluate changes in transmission intensity, fails to capture any specific information about the reservoir. As the ability of the parasite to persist by clonal antigenic variation (i.e. achieve a chronic infection) plays a key role in transmission from human to mosquito, we propose that the diversity of the genes underlying this phenotype provides a genetic fitness estimate of the reservoir, to complement existing molecular surveillance for drug resistance mutations [10], genes encoding vaccine antigens [11], [12], and microsatellite loci [8].

**Figure 1:**
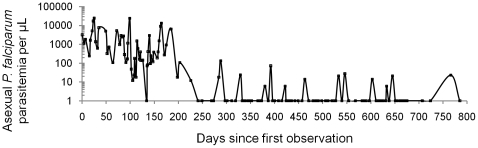
Clonal antigenic variation and parasite persistence. Asexual *P. falciparum* parasitemia followed over time in a naturally infected Puerto Rican child. The parasitemia follows a pattern of recurrent peaks that decline in amplitude with time. The parasitemia in this child, believed to be a clone, lasted nearly 800 days. These successive peaks of parasitemia are consistent with antigenically distinct waves of parasitemia in *P. falciparum* infection believed to be mediated by PfEMP1 that allow for parasite persistence [14]. The intra-host dynamics of parasitemia observed in semi-immune children [3] and induced human infections [49] are best explained by variant-specific immunity to PfEMP1 variants encoded by the *var* multigene family [50] rather than by immunity to single-copy antigen genes. Figure composed using data from [51].

*P. falciparum* Erythrocyte Membrane Protein-1 (PfEMP1), encoded by the *var* multigene family, is considered the major polymorphic surface antigen of the infected erythrocyte involved in clonal antigenic variation (reviewed in [13], [14]). PfEMP1, expressed during maturation by trophozoite (blood stage) and early gametocyte (transmission stage) parasites [15], mediates binding to endothelial cell receptors in the deep vasculature of host tissues [13], [14]. To achieve chronic infection by immune evasion, parasites periodically switch PfEMP1 isoforms displayed on the erythrocyte membrane by differential expression of up to 60 members of the *var* multigene family found throughout each parasite genome [13], [14], [16]. This process of clonal antigenic variation is believed to be responsible for parasite persistence and chronic infection seen in naturally infected individuals, as depicted in Figure 1 where a chronically-infected individual displays successive waves of parasitemia mediated by antigenic switching.

To characterize the reservoir of infection, we investigated the molecular epidemiology of the *var* multigene family in three *local* African parasite populations representing a range of epidemiologic characteristics (Table 1) with an expectation that we would see more *var* diversity in high transmission sites. *Bakoumba, Gabon* is a rural village of ∼3000 people in the South East in an area of meso- to hyperendemic *P. falciparum*. *Pikine, Senegal* is a suburb east of Dakar city with ∼770,000 inhabitants living in an area of hypoendemic malaria. *Kilifi, Kenya* is a rural district of ∼600,000 people on the East Coast, where malaria is hypo- to mesoendemic. *Var* gene diversity data were collected using a previously published population genomic framework [17]. We set out to determine i) the extent of *var* gene diversity in three African sites, ii) the level of *var* gene differentiation across the African continent, and iii) the population structure of *var* genes in Africa.

**Table 1:** Characteristics of the populations surveyed.

Survey Site	Date of collection	Annual EIR (infective bites/person/year)^a^	Human pop. size^b^	Clinical presentation	Age of individuals surveyed	Age-specific pop. size^c^	Age-specific *Pf* prevalence^d^	Age-specific mean MOI^e^	Total # of genomes in the age-specific pop.^f^
**Bakoumba, Gabon**	2000	102	3,000	Asymptomatic	6 months - 10 years	848	51.7%	2	877
**Pikine, Senegal**	2000–2005	0.014–0.86	768,826	Uncomplicated malaria, outpatient.	5 years and older	635,514	3.6%	1.42	32,487
**Kilifi, Kenya**	2002	0.0–53	350,000	Non-severe malaria, hospitalized	2 to 104 months	106,784	19%	2.32	47,070
**Amele, Papua New Guinea ** [**17**]	1999	44–293	5,300	Asymptomatic	6 months – 11 years	2153	37.4%	1.82	1466
**Porto Velho, Brazil ** [**19]**, [31****]	2001–2006	10	380,884	Asymptomatic to acute, non-severe	Not specified	380,884	0.46%	1.79	3136

Characteristics of the populations surveyed are listed in this table. For comparison, population characteristics corresponding to the Amele and Porto Velho published datasets are included. The population level data are estimated from published surveys and/or calculated as described:

a. Sources for annual Entomological Inoculation Rate (EIR): Bakoumba [22]; Pikine [24]; Kilifi [27]; Amele [52]; Porto Velho [53]. EIR reported as daily rates have been converted to annual rates by multiplying by 365. The Amele EIR estimate is specific to *P. falciparum* and excludes *P. vivax* infected bites.

b. Sources for population (pop.) estimates: Bakoumba [54]; Pikine (2002 Census) [55]; Kilifi (extrapolation based on demographic surveillance population) [56]; Amele (1987 survey) [57]; Porto Velho (2004 Census) [58].

c. Age-specific population sizes were estimated by multiplying the age-structured population frequencies by the total population size. Where the age-specific population in this study overlapped only partially with reported age groups, the proportion of overlap within the age group was used for the calculation. Country-level populations frequencies for Gabon, Kenya, and Senegal were calculated from reference [59]. For the Amele population, population frequencies were calculated from the age structure of the surveyed individuals in reference [60], reported to match the age structure of the population. For the Porto Velho, all age groups were included.

d. Sources for age-specific *P. falciparum (Pf)* prevalence estimates: Bakoumba [61]; Pikine [24]; Kilifi (2002 estimate) [56]; Amele [62]; Porto Velho [63].

e. Age-specific multiplicity of infection (MOI) estimates were derived from the following sources: Bakoumba [20]; Pikine [64] scoring multiple infections as double infections; Kilifi [65] simple average of the two sites; Amele [48] using all three markers; Porto Velho [66] using all three markers and counting multiple infections as double infections. These estimates of MOI reflect the age-range from which the samples were taken and are used to project the total circulating genomes in each population. They do not reflect the mean MOI for the study sites across all age groups.

f. Estimated total number of genomes in the age-specific population was calculated as:
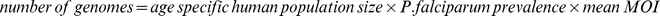

*Var* genes are composed of multiple Duffy-binding-like (DBL) and cysteine-interdomain-rich (CIDR) domains that adhere to host tissues to enhance parasite survival and contribute to pathogenesis [14]. DBLα, as the only domain found in nearly all *var* genes [13], is the most informative molecular marker of *var* gene diversity. We applied the sampling framework to survey the *var* DBLα domain in 29–30 natural *P. falciparum* isolates from each of three African populations. Using a degenerate PCR strategy, we randomly sampled *var* genes by amplifying, cloning, and sequencing approximately 100 copies of the DBLα domain from each isolate in the population samples [17]. Within each isolate, we eliminated redundancy (repeated sampling of the same *var* gene) by defining distinct *var* genes as those with less than 96% nucleotide identity to any other DBLα sequence. At the population level, we designated distinct ‘*var’* types by clustering the *var* genes, again using the 96% identity rule [17]. Previous studies have shown that DBLα sequences share on average ∼45–66% amino acid identity, and the vast majority of sequences share <75% amino acid identity (e.g. [17], [18]). We compared the diversity in African *var* DBLα sequence data with published DBLα data from two local, non-African populations: a cluster of Amele villages in Papua New Guinea (PNG) [17], and the municipality of Porto Velho (and suburbs), in the Western Amazonian state of Rondônia, Brazil [19].

## Materials and Methods

### Ethics Statement

Informed consent was obtained from all participants in the study. All data were analyzed anonymously. For the study in Gabon, ethical permission was obtained from the Ethics Committee for Research in Human Medicine, International Center for Medical Research, Gabon, and NYU School of Medicine Institutional Review Board. The informed consent procedure for the study consisted of a presentation of the aims of the study to the community. Individuals (or their parents) were asked to volunteer. At the time of collection, the purpose and design of the study were explained to each individual and verbal informed consent was collected by a minimum of two people. The verbal consent process was consistent with the ethical expectation at the time of enrollment. The ethics committees specifically approved this procedure. For the study in Senegal, ethical permission was obtained from the Ethics Committee of Cheikh Anta Diop University in Dakar and NYU School of Medicine Institutional Review Board. A verbal form of consent was chosen by the local scientific team as they considered it to be the most appropriate method of consent for this type of study. Verbal informed consent was obtained by the clinician reading the consent form to each participant. After verbal informed consent was obtained, the clinician signed and documented consent on the written consent form. The ethics committees specifically approved this procedure. For the study in Kenya, ethical approval was given by the Kenya Medical Research Institute (KEMRI) National Ethical Review Committee and NYU School of Medicine Institutional Review Board. Written consent was obtained from each participant.

### Study sites and populations

We compared three epidemiologically and geographically distinct *P. falciparum* populations of Central Africa (Bakoumba, Gabon), West Africa (Pikine, Senegal) and East Africa (Kilifi, Kenya). Bakoumba, Gabon isolates were collected from volunteers from the rural village of Bakoumba in the Haut-Ogooue Province [20]. This village consists of around 3000 people within a 10 km radius. Bakoumba is meso- to hyperendemic for *P. falciparum* with highly seasonal transmission. The entomological inoculation rate (EIR) during a peak transmission time has been measured as 0.83 infective bites/person/night [21], with an estimated mean EIR of 0.28 infective bites/person/night over a full year [22]. Isolates were collected from 604 children aged between 6 months to 10 years, presenting with non-clinical (asymptomatic) malaria in May 2000 [23]. We sampled *var* genes from 29 of these isolates.

Pikine, Senegal isolates were collected from individuals attending an outpatient clinic in Pikine, a suburb to the East of Dakar city. The population of Pikine near the time of the study was around 770,000 people living within a 10 km radius. In this area, malaria is hypoendemic with seasonal transmission between August and December, and an estimated annual EIR range of 0.014 to 0.86 infective bites/person/year (averages to a daily EIR of 0.00004 to 0.002 infective bites/person/night) [24]. Isolates were collected as part of malaria treatment studies from adults and children 5 years of age or greater presenting with symptoms consistent with mild malaria including fever, chills, and headache, and a positive malaria blood smear [25]. Samples were collected between 2000 and 2005. We sampled *var* genes from 29 of these isolates.

Kilifi, Kenya isolates were collected from individuals from the rural District of Kilifi, located in the Coast Province of Kenya. Kilifi District, at the time of the study, had a population of around 600,000 people living in rural villages within 75 km of each other. The estimated population from which the study participants came was around 350,000 people at the time of the study. Malaria is endemic in the area, with two annual peaks of transmission and subsequent disease in May to July, and in November [26]. Mean daily EIR varies by specific location, ranging from 0.000–0.145 infective bites/person/night [27]. Blood samples were taken from children (aged 2-104 months) admitted to the pediatric ward of the Kilifi District Hospital with a final diagnosis of non-severe malaria (no prostration, no respiratory distress, no prolonged seizures or coma). Samples were collected in 2002. We sampled *var* genes from 30 of these isolates.

We selected for inclusion in this study isolates identified as single-genome infections by genotyping of Merozoite Surface Protein 2 (*msp2*) [28]. This biased the investigation towards younger children in the study populations. In summary, Kilifi isolates were from hospitalized, non-severe clinical cases from a large rural catchment with transmission ranging from low to high; Pikine isolates were from outpatient, non-severe clinical cases from an urban population with low transmission; and Bakoumba isolates were from asymptomatic, non-clinical cases from a smaller, rural catchment with high transmission. Together, these populations represent in a wide range of malaria epidemiologies in Africa.

### DNA extraction and genotyping

Genomic DNA for each isolate was obtained by extraction of dried blood spots or whole blood with the QIAamp DNA Blood Mini Kit (Qiagen) according to the manufacturer's instructions. Isolates used for *var* gene sampling were identified as single genotype infections by genotyping of *msp2* using PCR amplification and restriction enzyme digestion [28]. In addition, we genotyped each isolate at 12 microsatellite repeat loci amplified by PCR with fluorescent-labeled primers [29]. Microsatellite alleles were differentiated by DNA fragment length as determined on an ABI 3700 sequencer and scored using GeneMapper software (Applied Biosystems). Some field isolates used in the study contained two genotypes when both methods were used. These methods confirmed that no two African isolates in the study were genotypically identical.

### High throughput *var* DBLα sequencing of African isolates

*Var* gene sampling based on the DBLα domain was performed as described elsewhere [17]. Briefly, DBLα domains were amplified from individual isolate genomic DNA samples using degenerate primers to blocks B, D, and/or H [30]. For eight of the 29 Bakoumba isolates, DBLα domains were amplified using primers to blocks D and H (Fwd-AGRAGYTTYGCNGAYATHGG Rev-AACCAYCTYAARTAYTGNGG) with PCR conditions as published [17]. For the remaining 21 Bakoumba isolates, DBLα domains were amplified using primers to blocks B and H (Fwd-GCMTGYGCDCCRTWYMGAMG Rev-TCKGCCCATTCYTCRAACCA; primer sequences provided by J. Smith). PCR conditions for these primers were as follows: 2 µl isolate genomic DNA, 1x Ampli Taq Gold reaction buffer (Applied Biosystems), 2.5 mM MgCl_2_, 0.15 mM dNTPs, 20 pmoles of each primer, 2.5 Units Ampli Taq Gold (Applied Biosystems). PCR cycling was carried out on a Perkin Elmer 9600 thermal cycler and involved 1 cycle of 95°C for 10 mins; 35 cycles of 95°C for 5 secs, 50°C for 20 secs and 60°C for 45 secs; 1 cycle of 60°C for 2 mins. For the Kilifi and Pikine isolates, DBLα domains were amplified using the primers to blocks B and H noted above. PCR conditions were 1 µl genomic DNA, 0.15 mM dNTPs, 2.5 mM MgCl_2_, 20 pmoles of each primer, 2.5 units Hotmaster Taq DNA Polymerase (Eppendorf). Reactions were performed in an Eppendorf EP Gradient Mastercycler thermal cycler at 94°C for 2 min; 35 cycles of 94°C for 5 sec, 50°C for 20 sec, and 60°C for 45 sec; and a final cycle at 60°C for 2 min. PCR bands of between 450–700 bp were purified using agarose gel electrophoresis and extraction with the Qiaquick Gel Extraction kit (Qiagen) according to manufacturer's instructions. PCR products were cloned and at least 96 clones sequenced with Universal forward and reverse primers as described previously [17]. Sequences from Gabon have been deposited in GenBank under the following accession numbers: DQ134044-6; DQ134086-92; DQ134281-2; DQ134514-5181; DQ135183-232; DQ135243-8; DQ135261-8; DQ135349-51; DQ135354; DQ135382; DQ135400; DQ135406-7; DQ135409-22; DQ135426-41; DQ135444-48; DQ135460-597. Sequences from Kilifi and Pikine have been deposited under the accession numbers HQ732288-HQ733853.

### *Var* sequence data from Papua New Guinea and Brazil

*Var* sequence data from all 30 isolates comprising the ‘local population’ from Amele, Papua New Guinea [17] were downloaded from GenBank. Accession numbers for downloaded Amele sequences are listed in Table S1. *Var* sequence data from 42 isolates from Porto Velho (and suburbs), Brazil [19], [31] were downloaded from the supplementary online file Supplemental Table 4 (applic6.txt) in [19]. Of the 43 Porto Velho isolates described [19], we excluded isolate S20 collected in 1985 and included the remaining 42 isolates collected from 2001–2006. A list of the 42 isolates included in the analysis can be found in Table S2. These sequence data encompass a region spanning the semi-conserved blocks D and H of the DBLα domain.

### *Var* sequence analysis

We have previously published our framework for analyzing *var* sequence data [17]. For comparability, block B-H *var* sequences were trimmed at the 5′ end before analysis so that only the block D-H region was compared. Quality controlled *var* gene sequences were aligned at 96% sequence identity using Sequencher 4.6 software (Genecodes) to remove redundancy among the reads from each isolate. We classified *var* sequences into distinct types through alignment using Sequencher 4.6 with a 96% sequence identity cutoff to identify matching sequences among isolates.

### Microsatellite Analysis

Twelve microsatellite markers were genotyped in *P. falciparum* isolates from Kilifi, Kenya (n = 47) Pikine, Senegal (n = 30); Bakoumba, Gabon (n = 24) were genotyped as previously described [29]. The isolates used for microsatellite analysis included 30 Kilifi isolates, 29 Pikine isolates, and 24 Bakoumba isolates used in the *var* gene analysis. Microsatellite haplotypes were defined for each parasite isolate and used for population genetic analysis. Microsatellite alleles were scored using GeneMapper software (Applied Biosystems). For the population genetic analyses of microsatellites we used the software program FSTAT [32].

### Cumulative diversity curves

Smoothed cumulative diversity curves, analogous to species accumulation curves, were plotted on Microsoft Excel with averaged data generated from EstimateS 8.0 software [33] set to 50 runs.

### Richness estimates

Non-parametric statistical estimates of richness, Chao1 and Abundance-based Coverage Estimator (ACE), and 95% confidence intervals were calculated using EstimateS 8.0 [33]. The Chao1 statistic [34] estimates the total number of types in a population using frequency data on types seen once only and types seen twice only. While the Chao1 estimator relies on singleton and doubleton data to estimate unseen species, the ACE estimator [35] utilizes all frequency data, divided into abundant types and rare types. We applied a cutoff value for rare groups of 10 as suggested [36]. Chao1 estimates were derived as a lower-bound estimate of richness, whereas ACE better approximates a point estimate of richness [36]. In the setting of equal probability of distribution and sampling of types, the Chao1 estimator yield a point estimate of richness. These estimators cannot predict a probable maximum richness. Curves depicting the stability of richness estimates with sample size were calculated using EstimateS [33].

### Frequency plots and rank abundance curves

Frequency plots and rank abundance curves [37] were tabulated and plotted using Microsoft Excel.

### Estimates of the number of genomes in survey populations

An approximation of the number of circulating genomes in a survey population was calculated as follows:
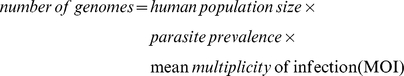
In this case, the survey population refers to the age-specific population from which *P. falciparum* isolates were taken, and the population size reflects the estimated population of that age group. This survey population size does differentiate between symptomatic and asymptomatic individuals, but rather includes all individuals of the specified age range. Population size estimates for each geographic unit were obtained from multiple sources as indicated in the Table 1. These population sizes were then adjusted to reflect the age-group specific population using published data on the age structure of each population. Sources for estimates of parasite prevalence and of MOI are referenced in Table 1. Data on parasite prevalence and MOI were selected to match to specific age groups and geographic locations surveyed where possible.

### Estimates of further sampling efforts

Using the ACE estimate of *var* type richness and a statistical predictor of the number of new species or types encountered in further sampling of a population [38], we estimated the proportion of *var* types in a population that will be identified given a range of sample sizes in each population. The model uses frequency information from ‘rare’ types, and we have used the suggested frequency cutoff of 10 for rare types [36]. The projections account for sampling of multiple infections in the population. Calculations were performed using the ‘Multinomial Model’ implemented in the software package SPADE [36], and plotted on Microsoft Excel.

## Results

### Sampling depth

**Figure 2:**
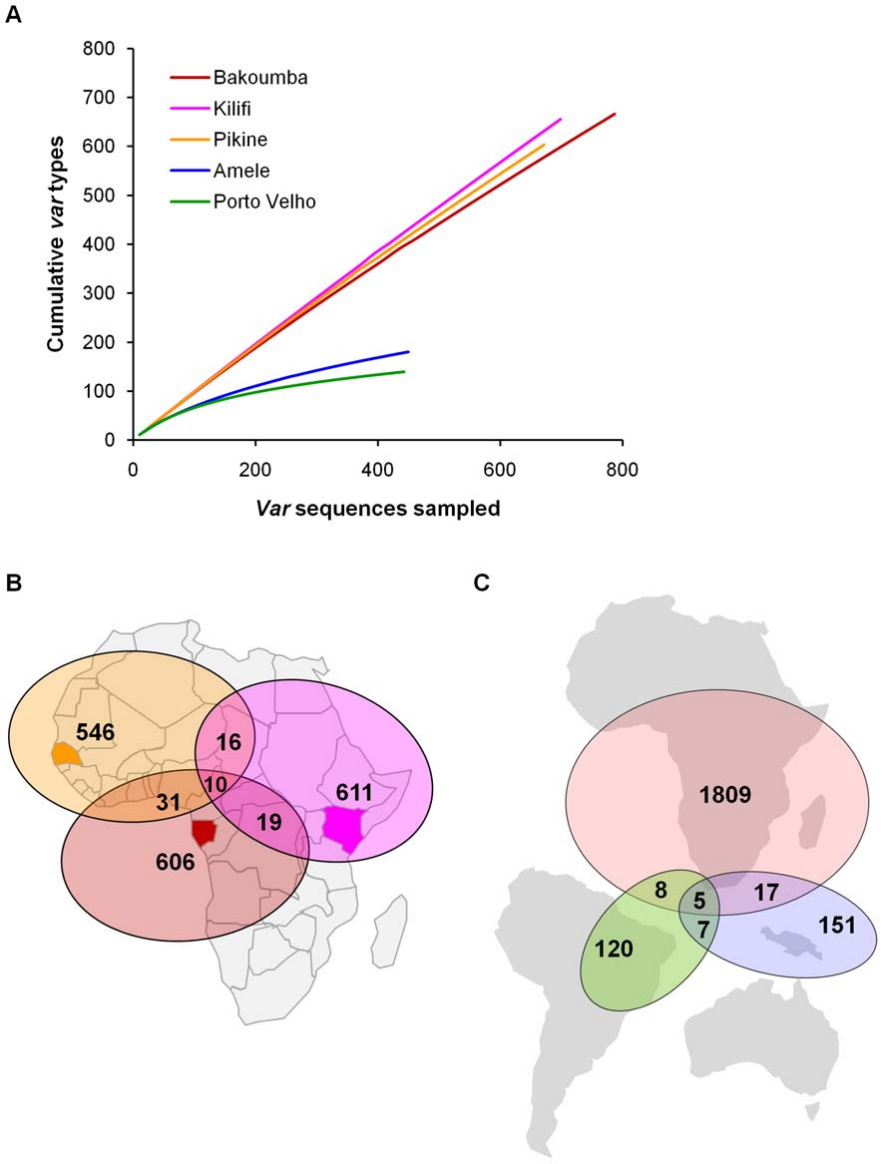
Diversity and sharing of *var* types among African and non-African populations. **A**) Cumulative diversity curves for each of the five populations. These averaged curves plot the cumulative number of *var* types observed with successive sampling of *var* sequences. A well-sampled population will show a curve that levels off and approaches an asymptote, that would approximate the total number of types in the population. The curves from the three African populations (Bakoumba, Pikine, Kilifi) did not show evidence of leveling off, in contrast to the curves from Amele, Papua New Guinea [17] or Porto Velho, Brazil [19]. Sampling of the African populations has not yet begun to approach the limits of diversity. **B**) Sharing of *var* types among the three African population samples. Between 26 and 41 *var* types were shared among any two population samples; only 10 *var* types were found in all three populations. **C**) The majority of *var* types in each continent were not found in the other continents. Only 5 *var* types were found in all three continents. Samples from Bakoumba, Pikine, and Kilifi represent Africa, samples from Amele represent Asia-Pacific, and samples from Porto Velho represent the Americas.

For each African isolate, the sampling yielded high quality DNA sequence data for a median of 83 (range 12–181) *var* clones. This sampling provided approximately 50% (median 24, range 3–47 unique *var* DBLα D–H tags) of the estimated 50–60 *var* genes per genome (Table 2). We measured depth of *var* gene sampling in each population with a cumulative diversity curve [17] depicting the rate at which new *var* types were identified with the collection of unique sequences from each isolate. Despite identifying ∼700 unique *var* sequences per African population, many *var* types remained undiscovered in each population as indicated by failure of the curves to plateau (Figure 2A). In contrast, sampling of the non-African populations of Amele and Porto Velho was more complete (Figure 2A).

**Table 2:** *Var* sampling and estimated *var* type richness in African and non-African populations.

Site	Genomes sampled	Unique *var* DBLα tags sampled	Observed *var* types	No. of *var* types found in only 1 isolate (%)	Chao1 richness estimate (95% CI)	ACE richness estimate	Proportion of total types sampled^a^
**Bakoumba, Gabon**	29	787	666	597 (90%)	4540 (3452–6053)	5557	15%
**Pikine, Senegal**	29	672	603	554 (92%)	5116 (3712–7156)	4844	12%
**Kilifi, Kenya**	30	699	656	622 (95%)	7565 (5292–10951)	8028	9%
**Amele, Papua New Guinea ** [**17**]	30	452	180	103 (57%)	369 (290–506)	370	49%
**Porto Velho, Brazil ** [**19]**, [31****]	42	443	140	59 (42%)	232 (186–332)	205	60%

For each population, the total number of genomes sampled, the non-redundant DBLα block D-H tags recovered, and the number of distinct *var* types identified are summarized. Estimates of richness (total *var* types) are listed using Chao1 and ACE estimators. Figures for Amele and Porto Velho were derived using published sequence data.

a. Calculated as follows: 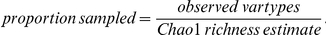

### Proportion of genomes surveyed

To determine the proportion of *P. falciparum* genomes surveyed in each population, we used parameter estimates from the literature to calculate the number of circulating *P. falciparum* genomes in each age-specific population:
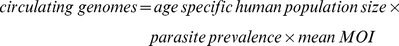



While population size and parasite prevalence varied greatly among populations, overall we sampled a minority of the circulating *P. falciparum* genomes in each population (Table 1). We estimated that in Bakoumba we sampled 29 of 877 circulating genomes (3.3%); in Pikine, 29 of 32487 genomes (0.09%); and in Kilifi, 30 of 47070 genomes (0.06%). Correspondingly, in Amele [17], 30 of an estimated 1446 parasite genomes (2.0%) were sampled, and in Porto Velho [19], 42 of an estimated 3136 genomes (1.3%) were sampled (Table 1).

### Estimates of *var* richness

Due to this shallow sampling, we applied statistical estimators designed for incompletely sampled data sets to estimate *var richness* (the total number of *var* types in the population). *Var* gene diversity was found to be high in all three African parasite populations using the Chao1 non-parametric statistical estimator, suitable for data sets with numerous rare types [34]. The richness estimates considered the age and clinical symptoms of each human population. We estimated 4540 *var* types in the Bakoumba population, 5116 types in Pikine, and 7565 types in Kilifi. In contrast, a log-order fewer *var* types were projected in the non-African populations: 369 in Amele (Papua New Guinea) and 232 in Porto Velho (Brazil). Projections using another non-parametric estimator (Abundance Coverage Estimator) yielded similar results (Table 2).

Both Bakoumba and Amele represent surveys of asymptomatic children in small (<10 km diameter), high transmission villages. A comparison of data from these two sites revealed considerably greater local *var* diversity specific to Africa. In Amele nearly half of the ∼400 projected *var* types were identified after sampling 30 genomes, whereas a similar sampling effort in Bakoumba yielded only 15% of the ∼4500 projected *var* types (Table 2). It follows that nearly a log-order more genomes must be sampled in Bakoumba compared to Amele to fully catalogue local *var* diversity (Figure S1). The references cited in Figure S1 and in other Supporting Information figures and tables are listed in Text S1.

### Geographic differentiation of *var* types and microsatellite markers

Minimal sharing of *var* types was observed among the three African populations (Figure 2B) suggesting spatial diversity. Of the 1839 African *var* types sampled, 66 (3.6%) were shared between any two sites and only 10 (0.5%) were found in all three sites. Genotyping of microsatellite repeat markers [29] in these samples demonstrated evidence of geographic differentiation at the genome-wide level between West Africa and East/Central Africa. Each parasite isolate had a unique microsatellite haplotype, but there was overlap among haplotypes (ie: matching alleles at one or more loci). The microsatellite diversity statistics are shown in Table S3. Analysis of the 3 African populations revealed no significant differentiation among the Kilifi and Bakoumba populations as measured by Wrights *F*_ST_ (*F*_ST_ = 0.019) (Table S4). However, Pikine and Kilifi populations were significantly differentiated (*F*_ST_ = 0.039; *P*<0.01) and Pikine and Bakoumba showed low levels of differentiation (*F*_ST_ = 0.019) which did not reach statistical significance. When grouped together, Kilifi and Bakoumba populations remained significantly differentiated from Pikine (*F*_ST_ = 0.033; *P*<0.01). While these data together suggest geographic differentiation of *var* genes within Africa, we have interpreted this observation with care as the paucity of shared *var* types may simply reflect shallow sampling in any site (Figure S2). Comparisons across continents revealed few shared *var* types between African sites and the well-sampled Amele or Porto Velho sites (Figure 2C) demonstrating larger scale geographic variation in *var* genes. Two of the shared *var* types have high sequence homology with the previously described unusual semi-conserved *var* gene *var1CSA* found in most parasite isolates [13] (Table S5).

### *Var* gene population structure

**Figure 3:**
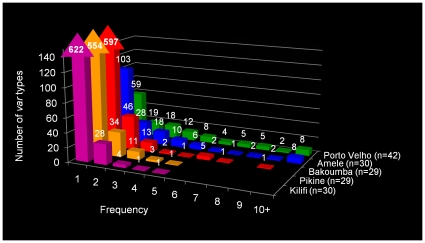
Frequency distribution of *var* types in the five population samples. Bakoumba, Kilifi, Pikine, Amele, and Porto Velho. In parenthesis next to each population is the number of isolates (n) sampled from the population. On the horizontal axis is the frequency class (in number of isolates) for each *var* type. The vertical axis depicts the number of *var* types found in each frequency class. For example, in the Kilifi dataset 622 *var* types were found in one isolate; 28 *var* types were each found in two isolates, etc. In the African populations, the overwhelming majority of *var* types were found in only one isolate. Differences in frequency distribution of *var* sequences were statistically significant by χ^2^ analysis (p<0.0001) (Table S6).

**Figure 4:**
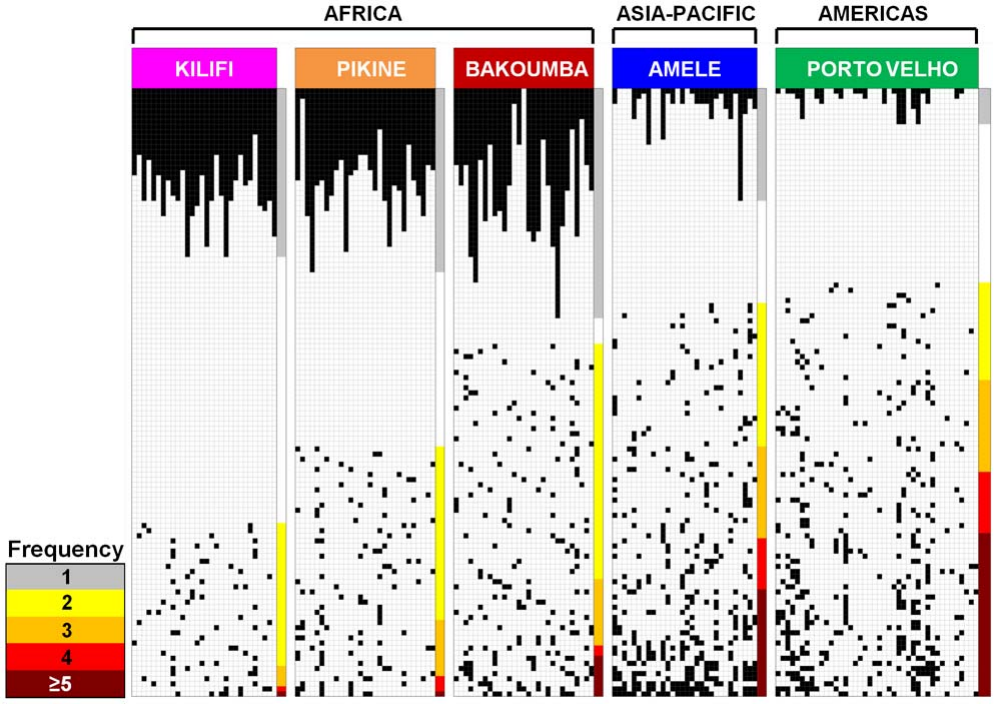
Organization of *var* genes in Africa (Bakoumba, Kilifi, Pikine), PNG (Amele) and Brazil (Porto Velho). Within each labeled population, columns represent individual parasite isolates, and the black boxes represent *var* genes found in that isolate. Black boxes at the top of Figure 4 represent rare *var* types (found only in one isolate). Black boxes in the lower portion of Figure 4 depict *var* types that were found in more than one isolate within a population. For *var* types found in more than one isolate, each row represents a distinct type within the population. The key at the bottom-left of Figure 4 depicts frequency (in number of isolates) with which a particular *var* type was found in the population sample. *Var* types found more frequently were placed towards the bottom of the figure. White space represents an unknown number of *var* types that were not sequenced. Note that the amount of whitespace is not associated with numbers of genes missing, but was necessary to demonstrate sharing among repertoires. There was greater sharing of *var* types among isolates in the non-African populations compared to the African populations. Among the African populations, there appears to be more *var* type sharing in the Bakoumba population.

The population structure of *var* genes in Africa, as defined by organization of *var* gene repertoires within and among isolates, differed greatly from that observed in PNG or Brazil (Figures 3 and 4). Within a local African population, the majority of *var* types were rare, i.e. identified in only one of the 29–30 isolates sampled. In contrast, there were fewer rare types and considerable overlap in *var* repertoires in local Amele and Porto Velho parasite populations. It is noteworthy that the three African populations showed similar patterns of abundance (predominantly rare types) (Figure S3) despite marked differences in transmission and epidemiology.

### Comparison of *var* diversity with malariometric indices

**Figure 5:**
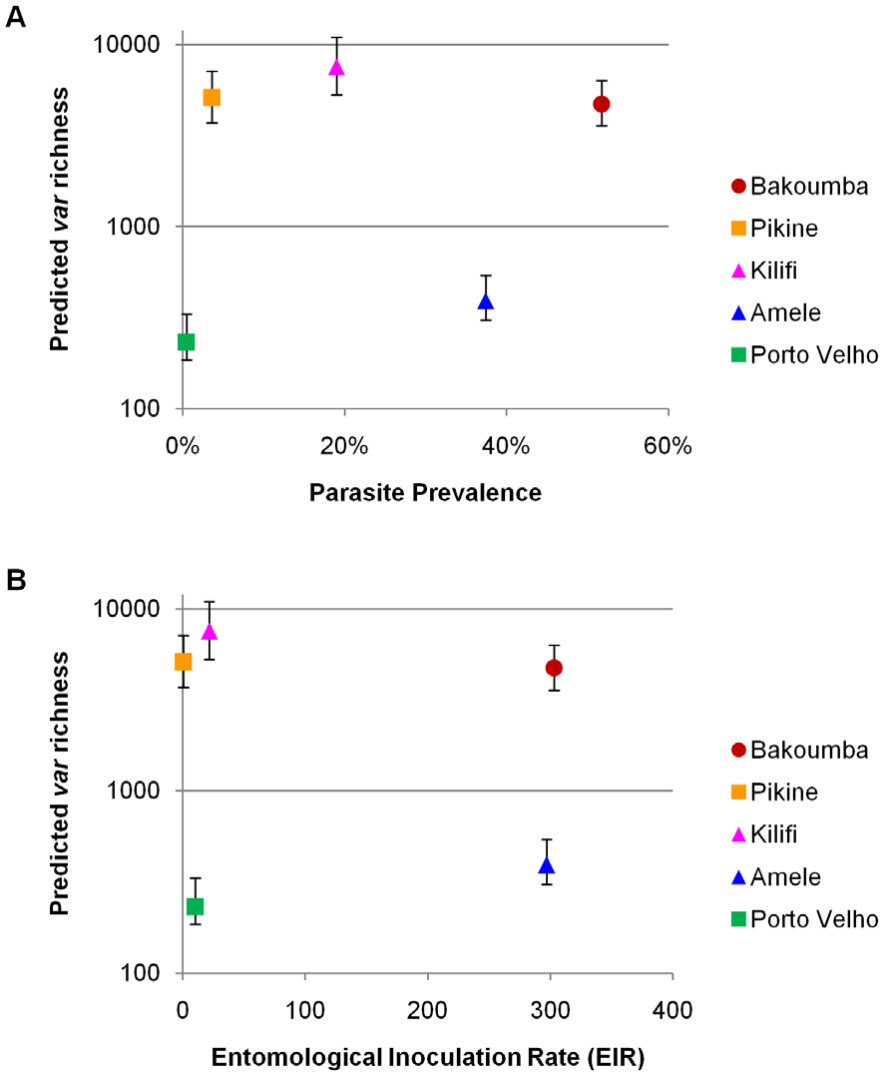
Relationship between *var* richness estimates and malariometric indices. For each population sampled, *var* richness estimated using the Chao1 equation is plotted against **A**) parasite prevalence and **B**) transmission as represented by entomological inoculation rate (EIR). We have used published parasite prevalence and EIR figures which are listed in Table 1. Where EIR has been reported as a range, we have plotted the midpoint of the range. The bars above and below each point represent the 95% confidence interval of the Chao1 estimate. Despite differences in transmission intensity and parasite prevalence, the local African populations all exhibited high estimates of *var* richness, roughly a log-order greater than the non-African populations.

*Var* diversity data was compared with standard malariometric indices such as prevalence of a positive blood smear and *entomological inoculation rate* (EIR), i.e. the number of bites by infectious mosquitoes received per person per year (Figure 5). We observed high *var* type richness throughout a range of parasite prevalence and transmission intensities in the African populations. Across continents, despite similar transmission levels and parasite prevalence in Amele and Bakoumba, large differences in *var* type richness were seen. Conversely, despite large differences in transmission and parasite prevalence, Amele and Porto Velho displayed similar *var* richness that was an order of magnitude lower than in the African populations. Strikingly, no correlation existed between *var* richness and prevalence, or *var* richness and EIR among the sites. The finding of high *var* type richness in low prevalence/low transmission settings such as Pikine demonstrates that *var* genetics can quantitatively discern complexity in the parasite reservoir.

## Discussion

The goal of any malaria elimination campaign is to reduce the reservoir of malaria infection in humans to zero locally. Success towards elimination would be monitored by measuring reductions in malaria transmission intensity using both EIR [7] and antigen-specific serological methods [39]. Reductions in the size of the reservoir of infection in humans would be monitored by blood smear positivity [7]. This measure is well known to be insensitive to changes in transmission intensity when parasite prevalence or rates reach 1 to 5% [7]. It also does not identify the extent of diversity of the reservoir nor the existence of multiple genomes per host. Similarly, more sensitive species-specific diagnostic methods currently in development will not capture this information. Current efforts in malaria genomics have neglected to analyze the reservoir of infection.

Here we present the first study to explore the reservoir of infection in *local* parasite populations, through the lens of the diversity of the major surface antigen of the blood stage of *P. falciparum*. We have focused on the *var* genes as a measure of parasite fitness due to their role in both establishing chronic *P. falciparum* infections and promoting transmission to the mosquito. Our choice of this antigen fits the well-established microbiological paradigm that global surveillance of any antigenically diverse pathogen (e.g Influenza A, HIV-1) focuses on documenting diversity of the major surface antigen involved in immune evasion.

A detailed analysis of *var* genetics within local African sites compared to previously published data from local sites in PNG and Brazil was completed. Analysis of 2158 unique *var* sequences from 88 African isolates did not reveal the limit to *var* richness. We estimate *var* richness in each local African population to be five to seven thousand types. These are likely to be underestimates for several reasons including limited sampling (Figure S4); Chao1 estimates representing a lower bound of richness [34]; greater diversity when entire *var* genes are analyzed; and observed fine spatial heterogeneity such as that seen in plant communities [40] resulting in non-representative sampling. The richness may be even higher within the general population of each site if the *var* types seen within an age-restricted subset differ from those in the population at large. Data presented in Figure 5 show that the depth of diversity affords greater sensitivity to define the reservoir of infection and measure incremental reductions in the size of the reservoir with control, particularly in the African sites, compared to parasite prevalence. Clearly, this extensive variation presents an opportunity for parasite surveillance, although a major challenge to vaccine design.

The genomic *var* diversity data presented complement those previously reported as our study has focused on *local* parasite populations. We showed in the past that high worldwide *var* diversity existed by comparing Amele (a local PNG population) with a global dataset [17]. Herein we describe high *var* diversity in *local African* populations, greatly exceeding that in local PNG or South American populations by at least an order of magnitude. Supporting our observations, others have described extensive local African *var* diversity in studies of severe malaria, although these studies mostly analyzed transcribed *var* genes rather than genomic *var* genes that constitute the reservoir (e.g. [41], [42]). The extent of *var* diversity in local African parasite populations compared to PNG and South America is consistent to that seen for genome-wide SNPs, microsatellites and mitochondrial genome diversity data, albeit with greater diversity in the *var* system [8], [43], [44]. This is best explained by the long population history and documented high recombination rates of *P. falciparum* in Africa [44].

The observed spatial diversity in *var* types warrants follow up as such geographic variation could be extremely important in the maintenance of diversity, as is the case for plant species [40]. How *var* gene variation exists in space and time needs to be elucidated by appropriate sampling and next-generation sequencing. In addition, the observed small scale spatial diversity of *var* genes suggests that *var* genetics could greatly inform current malaria mapping approaches [45] as well as predict complex malaria population dynamics due to the import of *var* types to areas where there is no widespread pre-existing immunity in the population.

Presently, limited *var* gene data are available from the *P. falciparum* genome sequencing projects, and planned 3X coverage for the majority of genomes is insufficient for assembly of the recombinogenic telomeric regions where most *var* genes reside [46]. Despite next-generation sequencing methods, *var* genes have largely been ignored due to these assembly issues. This seems extraordinary given their importance for parasite survival. Our African data suggest that we would need to sequence the *var* genes of hundreds of genomes to observe the full extent of *var* DBL*a* diversity in one local population (Figure S1). These data also establish the scale of the next generation sequencing effort and the requirement for high density spatial sampling in a few endemic areas.

Figure 3 and 4 present the first comparative analysis of the population structure of the *var* multigene family in multiple local populations. Despite different epidemiologies in the African populations, *var* type frequency distributions (Figure 3) and levels of repertoire overlap (Figure 4) appeared essentially the same but significantly different from local PNG and South American populations. Indeed, negligible *var* repertoire overlap was seen within any of three local African settings indicating that reinfected individuals would be more likely to encounter parasites with repertoires of *var* types to which they lack immunity. We propose that this lack of *var* repertoire overlap in parasite genomes within each African site favors superinfection and persistence in a semi-immune host. This observation can explain why semi-immune African children harbor multiple parasite genomes (peak mean MOI of 5 in children 3–7 years old) and the number of genomes reduces as children become more immune [5].

Immense *var* gene diversity and the observed lack of repertoire overlap in Africa pose a great challenge to malaria control and elimination in that immunity to infection only develops after many exposures which may be due to the numerous (antigenically distinct) *var* types in each population. Furthermore, the extent of overlap among the *var* repertoires will determine cross-immunity in the host population to distinct parasite genomes. Consequently, levels of cross-immunity would be higher in PNG and South American sites compared to African sites. Finally, the low repertoire overlap in Africa demonstrates the enormous potential for outcrossing in these parasite populations [47], [48] to quickly generate further genomic diversity of *var* repertoires unless elimination strategies are implemented and sustained.

We observed high *var* diversity in both low and high prevalence sites in Africa. This striking lack of correlation between prevalence and *var* diversity shows that *var* typing provides a complementary measure of the reservoir of infection to parasite prevalence. We propose that this molecular epidemiological surveillance system will prove particularly useful in monitoring incremental changes in the population structure and size of the reservoir of infection at low parasite prevalence in the new era of malaria eradication.

Thus, we conclude that *var* population genomics provides new insights into the epidemiology of *P. falciparum* in Africa where malaria has never been conquered. In particular, we have been able to describe the nature and extensive size of the reservoir of infection in local African sites as well as discover a unique *var* population structure that can facilitate superinfection with minimal overlap among *var* repertoires.

## Supporting Information

Figure S1 **The yield of further** ***var*** **sampling efforts.** The predicted proportion of total types observed was plotted as a function of sample size, using a non-parametric estimator [1] modeled on data from Bakoumba, Gabon and Amele, Papua New Guinea. Applying our present framework for sampling, we would need to sample 320 isolates to achieve 80% coverage of *var* types in the surveyed age group in Bakoumba, Gabon. In contrast, in Amele, PNG a sample of 40 isolates would suffice to achieve similar coverage. It is clear that to achieve similar coverage of *var* types in Africa and PNG requires vastly greater sampling effort in African populations. These projections were based on sampling of all parasitemic individuals, and not limited only to those with single clone infections. Application of next generation sequencing technologies may result in a higher yield of *var* sequences per isolate, and decrease the total number of isolates needed; however sampling in Africa would still require much greater number of isolates as compared to PNG.
(TIF)

Figure S2 **Potential effect of sampling depth on appearance of differentiation.** Hypothetical sampling of three identical populations, each containing the same highly diverse set of 1000 distinct *var* types. A) A random sample of 10% (100 types) is taken from each of the three populations. B) Assuming equal probability of sampling each *var* type, the resulting 10% samples from each population would demonstrate few *var* types shared between populations, even though the composition of each population is identical. C) A random sample of 80% (800 types) is taken from each of the three populations. D) If 80% of each population is sampled, the samples would display greater sharing of *var* types and would more closely resemble the true relationship among the populations (D). This exercise depicts one scenario where shallow sampling of populations may give the appearance of differentiation. Outcomes will vary with differences in total diversity, distribution of diversity, and sharing in the populations compared.
(TIF)

Figure S3 ***Var*** **rank abundance of African and non-African populations.** For each population, the relative abundance of each *var* type was plotted against the abundance rank of that *var* type. Relative abundance refers to the proportion of the total *var* sequences in the population sample. The slope of the curve reflects the evenness of the relative abundances of the *var* types sampled. A steep curve, as seen in Amele, denotes a more heterogeneous distribution of types.
(TIF)

Figure S4 **Stability of richness estimators with sample size.** In this figure, averaged Chao1 and ACE richness estimates were plotted against the number of *var* genes sampled for each population. Estimates of richness for the African populations (Kilifi, Pikine, Bakoumba) were a log-order of magnitude greater than those of the S. American (Porto Velho) or PNG (Amele) populations. Richness estimates demonstrated stable increases (slope of curve) with greater sampling [2].
(TIF)

Table S1 **List of accession numbers for Amele sequences used in the analysis.** GenBank Accession numbers for the 460 Amele DBLα sequences used in this analysis [3].
(DOC)

Table S2 **List of Porto Velho isolates used in the analysis.** DBLα sequences from 42 Porto Velho isolates [4], [5] used in this analysis. Sequence data was downloaded from the supplementary online file Supplemental Table 4 (applic6.txt) in reference [4].
(DOC)

Table S3 **Allelic diversity of African local populations based on analysis of 12 microsatellite loci.** Kilifi was the most diverse parasite population, followed by Bakoumba and Pikine. n  =  number of samples, SE  =  standard error.
(DOC)

Table S4 **Geographic differentiation of African local populations based on analysis of 12 microsatellite loci.** Wrights FST values for population comparisons by microsatellite alleles calculated using FSTAT [6].
(DOC)

Table S5 **African** ***var*** **types shared across populations.** From the datasets analyzed, this table lists the African *var* types also found in Amele and/or Porto Velho, as well as *var* types found in all three African populations. Under ‘Accession Number,’ a representative African sequence is listed. The table indicates the populations in which each type was found. Two shared *var* types, DQ135232 and DQ135587, demonstrated high homology to *var1CSA*, an unusual semi-conserved *var* gene [7]. No common sequences matched the conserved *var* gene *var2csa,* however the primers were not designed to amplify this unique gene.
(DOC)

Table S6 **Chi-square test on the distribution** ***var*** **sequence frequencies among the five populations.** Differences in frequency distribution among the five populations are statistically significant by χ2 analysis (χ2 = 1152; *p*<0.0001).
(DOC)

Text S1 **Supporting references.**
(DOC)
