# Supplementary figures and images for: A Molecular Epidemiological Study of var Gene Diversity to Characterize the Reservoir of Plasmodium falciparum in Humans in Africa
Source: PLoS One. 2011 Feb 9;6(2):e16629. doi: 10.1371/journal.pone.0016629 (PMC3036650; doi:10.1371/journal.pone.0016629)

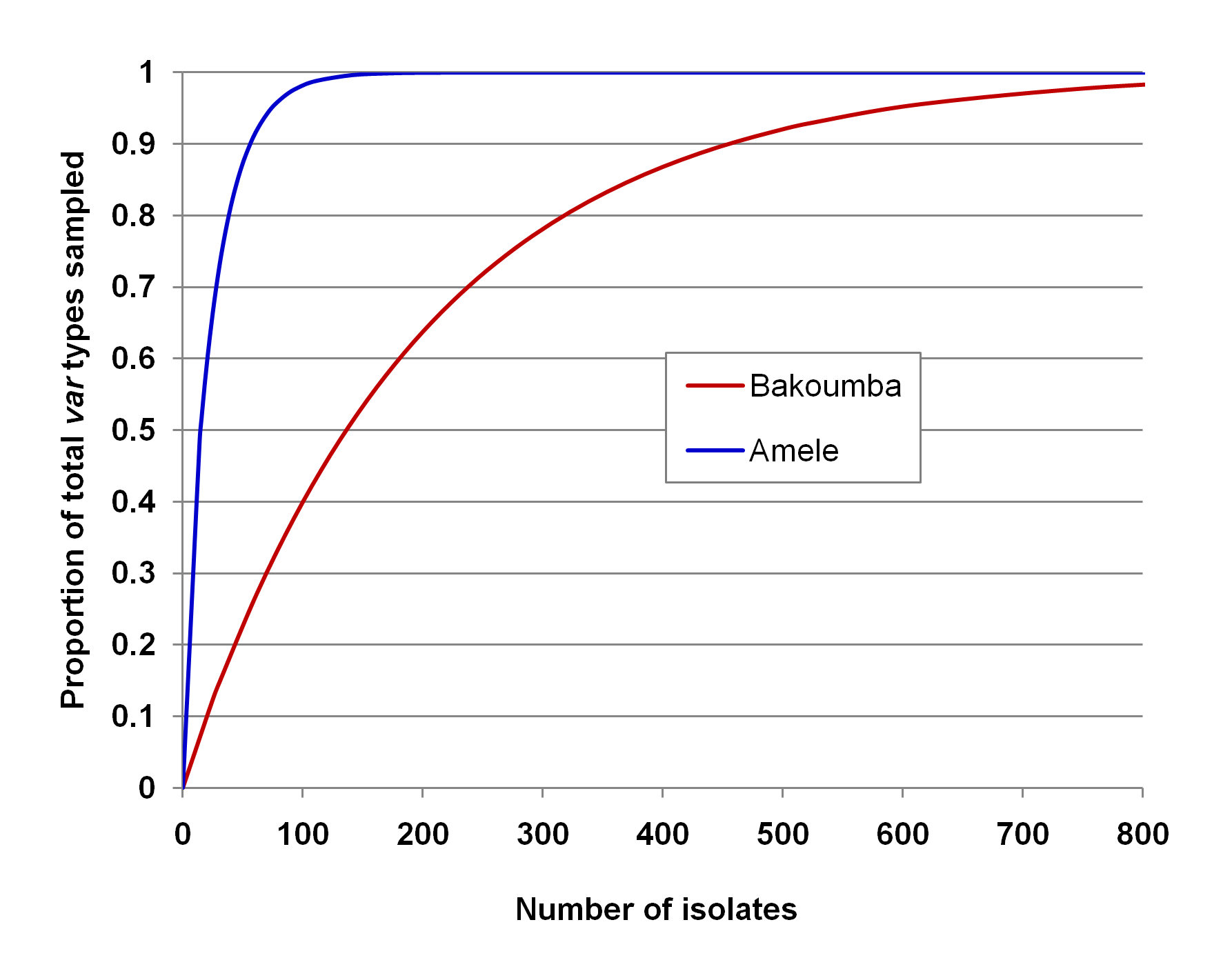

Supplement: Figure S1 — The yield of further var sampling efforts. The predicted proportion of total types observed was plotted as a function of sample size, using a non-parametric estimator [1] modeled on data from Bakoumba, Gabon and Amele, Papua New Guinea. Applying our present framework for sampling, we would need to sample 320 isolates to achieve 80% coverage of var types in the surveyed age group in Bakoumba, Gabon. In contrast, in Amele, PNG a sample of 40 isolates would suffice to achieve similar coverage. It is clear that to achieve similar coverage of var types in Africa and PNG requires vastly greater sampling effort in African populations. These projections were based on sampling of all parasitemic individuals, and not limited only to those with single clone infections. Application of next generation sequencing technologies may result in a higher yield of var sequences per isolate, and decrease the total number of isolates needed; however sampling in Africa would still require much greater number of isolates as compared to PNG. (TIF) [file pone.0016629.s001.tif]

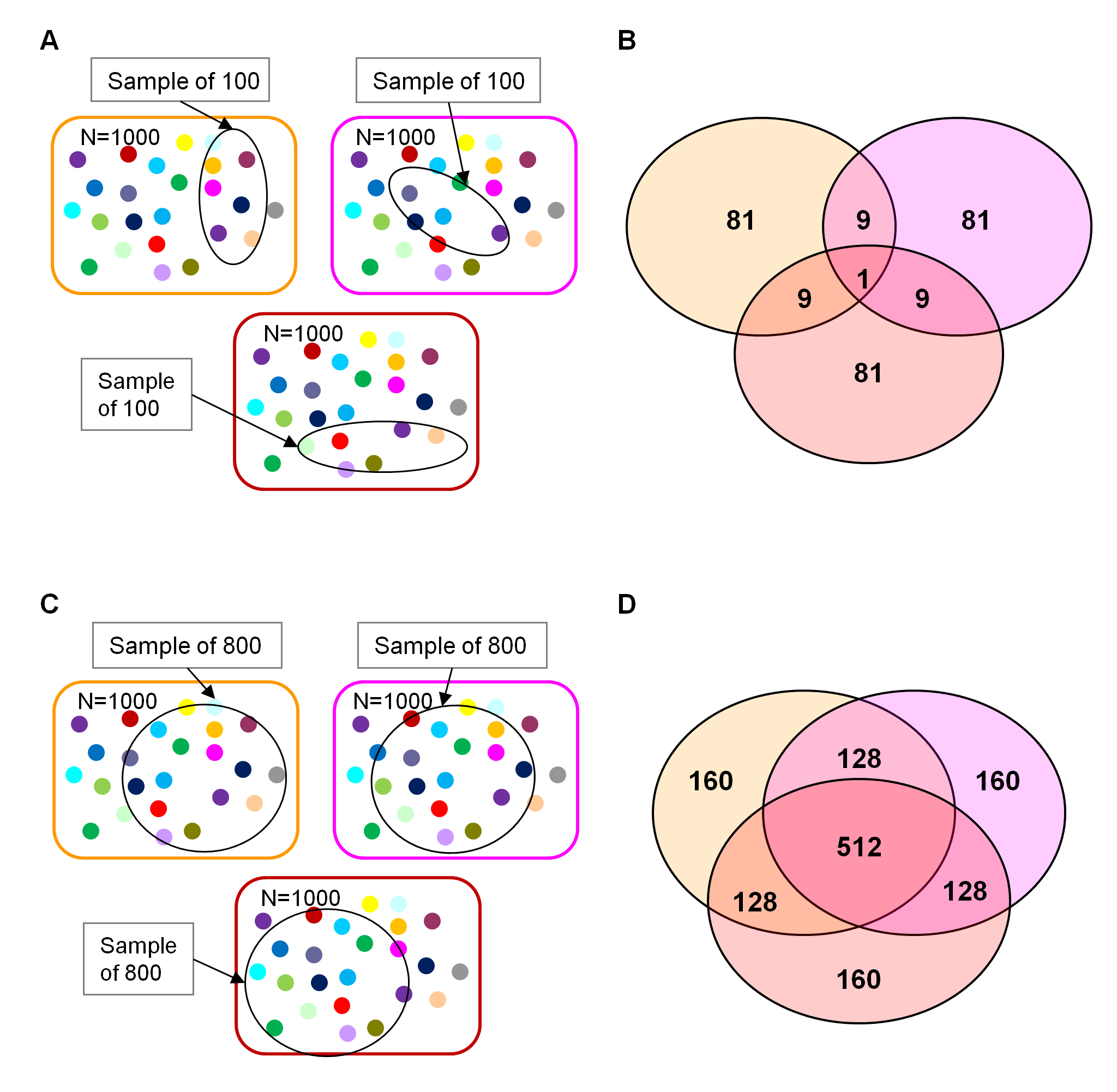

Supplement: Figure S2 — Potential effect of sampling depth on appearance of differentiation. Hypothetical sampling of three identical populations, each containing the same highly diverse set of 1000 distinct var types. A) A random sample of 10% (100 types) is taken from each of the three populations. B) Assuming equal probability of sampling each var type, the resulting 10% samples from each population would demonstrate few var types shared between populations, even though the composition of each population is identical. C) A random sample of 80% (800 types) is taken from each of the three populations. D) If 80% of each population is sampled, the samples would display greater sharing of var types and would more closely resemble the true relationship among the populations (D). This exercise depicts one scenario where shallow sampling of populations may give the appearance of differentiation. Outcomes will vary with differences in total diversity, distribution of diversity, and sharing in the populations compared. (TIF) [file pone.0016629.s002.tif]

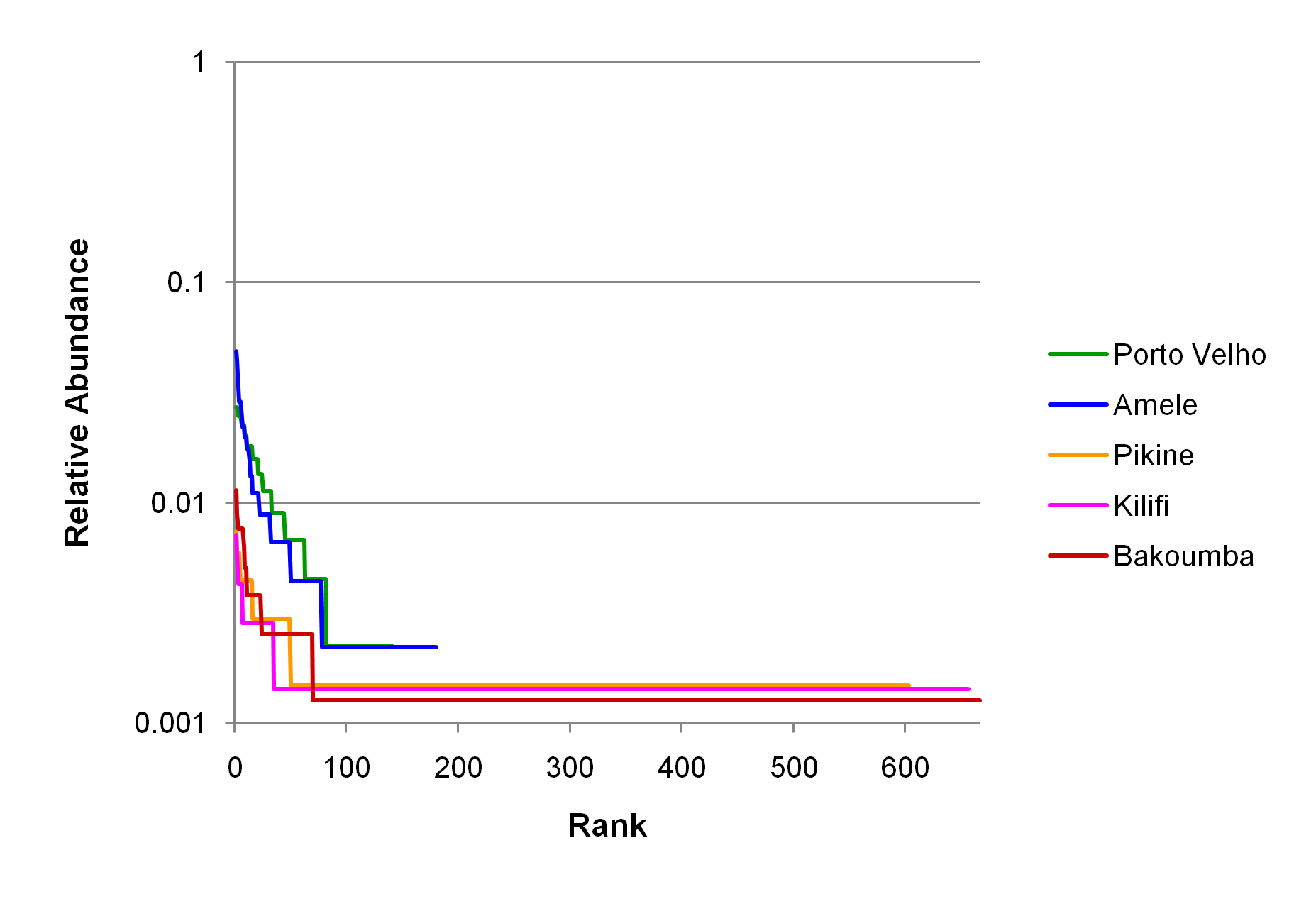

Supplement: Figure S3 — Var rank abundance of African and non-African populations. For each population, the relative abundance of each var type was plotted against the abundance rank of that var type. Relative abundance refers to the proportion of the total var sequences in the population sample. The slope of the curve reflects the evenness of the relative abundances of the var types sampled. A steep curve, as seen in Amele, denotes a more heterogeneous distribution of types. (TIF) [file pone.0016629.s003.tif]

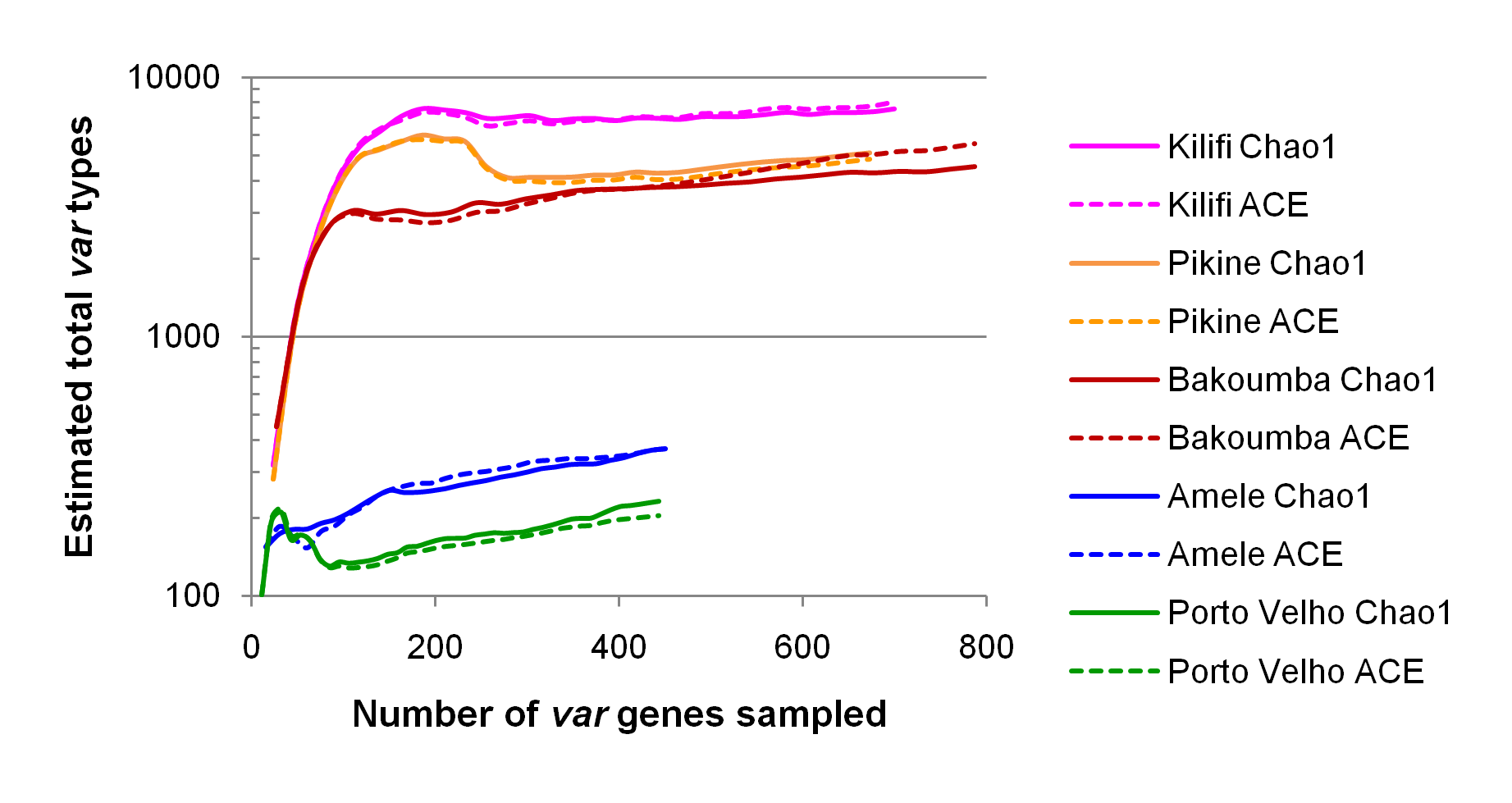

Supplement: Figure S4 — Stability of richness estimators with sample size. In this figure, averaged Chao1 and ACE richness estimates were plotted against the number of var genes sampled for each population. Estimates of richness for the African populations (Kilifi, Pikine, Bakoumba) were a log-order of magnitude greater than those of the S. American (Porto Velho) or PNG (Amele) populations. Richness estimates demonstrated stable increases (slope of curve) with greater sampling [2]. (TIF) [file pone.0016629.s004.tif]
