# Supplementary material for: A Molecular Epidemiological Study of var Gene Diversity to Characterize the Reservoir of Plasmodium falciparum in Humans in Africa
Source: PLoS One. 2011 Feb 9;6(2):e16629. doi: 10.1371/journal.pone.0016629 (PMC3036650; doi:10.1371/journal.pone.0016629)
Supplement: Table S1 — List of accession numbers for Amele sequences used in the analysis. GenBank Accession numbers for the 460 Amele DBLα sequences used in this analysis [3]. (DOC) [file pone.0016629.s005.doc]

**Table S1**

| DQ134042  DQ134074  DQ134138  DQ134164  DQ134177  DQ134180  DQ134183  DQ134195  DQ134373  DQ134435  DQ135331  DQ135339  DQ135341  DQ135353  DQ135373  DQ135820  DQ134057  DQ134100  DQ134109  DQ134119  DQ134200  DQ134239  DQ134247  DQ134251  DQ134261  DQ134266  DQ134274  DQ134295  DQ135182  DQ135251  DQ135253  DQ135318  DQ135377  DQ135842  DQ135843  DQ135844  DQ135845  DQ135846  DQ134141  DQ134154  DQ134169  DQ134186  DQ134204  DQ134221 | DQ134232  DQ134318  DQ134325  DQ134326  DQ134329  DQ134336  DQ134338  DQ134342  DQ134378  DQ134440  DQ135329  DQ134058  DQ134110  DQ134120  DQ134201  DQ134240  DQ134252  DQ134262  DQ134267  DQ134278  DQ135389  DQ135458  DQ135847  DQ135848  DQ134059  DQ134121  DQ134253  DQ134271  DQ134284  DQ134508  DQ135849  DQ134060  DQ134122  DQ134202  DQ134241  DQ134254  DQ134268  DQ134275  DQ134279  DQ134285  DQ134355  DQ134509  DQ135269  DQ135357 | DQ135385  DQ135391  DQ135393  DQ135850  DQ134061  DQ134101  DQ134123  DQ134203  DQ134242  DQ134248  DQ134255  DQ134276  DQ134280  DQ134286  DQ134291  DQ134296  DQ134303  DQ134305  DQ134313  DQ134352  DQ134415  DQ134510  DQ135851  DQ135852  DQ135853  DQ135854  DQ135855  DQ134062  DQ134077  DQ134124  DQ134142  DQ134155  DQ134170  DQ134205  DQ134233  DQ134243  DQ134272  DQ134297  DQ134306  DQ134345  DQ134348  DQ134371  DQ134447  DQ134456 | DQ135369  DQ135379  DQ135399  DQ135408  DQ135856  DQ135857  DQ134063  DQ134102  DQ134125  DQ134206  DQ134244  DQ134256  DQ134263  DQ134287  DQ134292  DQ134314  DQ134511  DQ134587  DQ135364  DQ135395  DQ135858  DQ135859  DQ135860  DQ135861  DQ135862  DQ135863  DQ135864  DQ135865  DQ135866  DQ134064  DQ134126  DQ134207  DQ134245  DQ134249  DQ134257  DQ134269  DQ134288  DQ134315  DQ134353  DQ134356  DQ134512  DQ135867  DQ134078  DQ134128 | DQ134143  DQ134156  DQ134171  DQ134187  DQ134209  DQ134307  DQ134319  DQ134330  DQ134339  DQ134359  DQ134363  DQ134365  DQ134368  DQ134372  DQ134374  DQ134379  DQ134384  DQ134391  DQ134401  DQ134443  DQ134451  DQ135335  DQ135387  DQ135868  DQ135869  DQ135870  DQ134050  DQ134069  DQ134096  DQ134134  DQ134408  DQ134463  DQ134588  DQ134065  DQ134127  DQ134208  DQ134072  DQ134103  DQ134129  DQ134144  DQ134157  DQ134188  DQ134210  DQ134222 | DQ134258  DQ134293  DQ134308  DQ134320  DQ134331  DQ134357  DQ134366  DQ134380  DQ134385  DQ134396  DQ134399  DQ134402  DQ134406  DQ134409  DQ134413  DQ134416  DQ134418  DQ134421  DQ134431  DQ135371  DQ135871  DQ135872  DQ134131  DQ134146  DQ134159  DQ134212  DQ134309  DQ134322  DQ134130  DQ134145  DQ134158  DQ134172  DQ134189  DQ134211  DQ134321  DQ134375  DQ134381  DQ134386  DQ134392  DQ134400  DQ134426  DQ134432  DQ135333  DQ134079 | DQ134132  DQ134147  DQ134160  DQ134190  DQ134213  DQ134332  DQ134382  DQ134433  DQ134452  DQ134454  DQ135235  DQ135348  DQ135381  DQ135873  DQ134080  DQ134133  DQ134148  DQ134161  DQ134191  DQ134214  DQ134360  DQ134369  DQ134393  DQ134403  DQ134427  DQ134436  DQ134441  DQ134444  DQ135213  DQ135345  DQ135874  DQ135875  DQ134162  DQ134215  DQ134081  DQ134163  DQ134216  DQ134448  DQ135876  DQ134066  DQ134082  DQ134104  DQ134149  DQ134166 | DQ134173  DQ134192  DQ134223  DQ134227  DQ134234  DQ134310  DQ134333  DQ134422  DQ134428  DQ134437  DQ134445  DQ134457  DQ134461  DQ134464  DQ135337  DQ135361  DQ134083  DQ134150  DQ134174  DQ134193  DQ134217  DQ134228  DQ134346  DQ134349  DQ134376  DQ134387  DQ134423  DQ134429  DQ134438  DQ134458  DQ134466  DQ135343  DQ134051  DQ134070  DQ134097  DQ134106  DQ134113  DQ134151  DQ134246  DQ134250  DQ134290  DQ134417  DQ134465  DQ135375 |
| --- | --- | --- | --- | --- | --- | --- | --- |

**Table S1, continued.**

| DQ135425  DQ135451  DQ135877  DQ135878  DQ135879  DQ134043  DQ134052  DQ134071  DQ134075  DQ134107  DQ134114  DQ134135  DQ134167  DQ134230 | DQ134238  DQ134420  DQ135283  DQ135402  DQ135443  DQ134115  DQ134116  DQ134139  DQ134152  DQ134165  DQ134168  DQ134178  DQ134181  DQ134184 | DQ134196  DQ134219  DQ134225  DQ134312  DQ134317  DQ134335  DQ134341  DQ134358  DQ134362  DQ134364  DQ134405  DQ134425  DQ134453  DQ134460 | DQ135359  DQ135821  DQ134583  DQ134586  DQ135209  DQ135211  DQ135212  DQ135214  DQ135240  DQ135405  DQ135822  DQ135823  DQ135824  DQ135825 | DQ135826  DQ135827  DQ135828  DQ135829  DQ135830  DQ135831  DQ135832  DQ135833  DQ135834  DQ135835  DQ135836  DQ135837  DQ135838  DQ135839 | DQ134053  DQ134054  DQ134055  DQ134098  DQ134108  DQ134117  DQ134302  DQ135215  DQ135241  DQ135367  DQ135840  DQ135841  DQ134056  DQ134099 | DQ134118  DQ134140  DQ134153  DQ134185  DQ134197  DQ134198  DQ134220  DQ134226 DQ134270  DQ134304  DQ134324  DQ134328  DQ134337  DQ134367 | DQ134383  DQ134390  DQ134395  DQ134398  DQ134412  DQ134076  DQ134199  DQ134231  DQ134344  DQ134347 |
| --- | --- | --- | --- | --- | --- | --- | --- |
