# Supplementary material for: A Molecular Epidemiological Study of var Gene Diversity to Characterize the Reservoir of Plasmodium falciparum in Humans in Africa
Source: PLoS One. 2011 Feb 9;6(2):e16629. doi: 10.1371/journal.pone.0016629 (PMC3036650; doi:10.1371/journal.pone.0016629)
Supplement: Table S2 — List of Porto Velho isolates used in the analysis. DBLα sequences from 42 Porto Velho isolates [4], [5] used in this analysis. Sequence data was downloaded from the supplementary online file Supplemental Table 4 (applic6.txt) in reference [4]. (DOC) [file pone.0016629.s006.doc]

**Table S2**

| **Isolate name** | **Isolate name** | **Isolate name** |
| --- | --- | --- |
| 16 | 42 | 114 |
| 17 | 51 | 115 |
| 18 | 91 | 116 |
| 19 | 97 | 134 |
| 20 | 98 | 135 |
| 21 | 99 | 383 |
| 22 | 101 | 405 |
| 23 | 102 | 429 |
| 30 | 103 | JO1 |
| 31 | 104 | JO2 |
| 32 | 105 | RDM17 |
| 33 | 106 | RDM42 |
| 34 | 112 | RDM61 |
| 35 | 113 | RDM62 |
