# Supplementary material for: A Molecular Epidemiological Study of var Gene Diversity to Characterize the Reservoir of Plasmodium falciparum in Humans in Africa
Source: PLoS One. 2011 Feb 9;6(2):e16629. doi: 10.1371/journal.pone.0016629 (PMC3036650; doi:10.1371/journal.pone.0016629)
Supplement: Table S3 — Allelic diversity of African local populations based on analysis of 12 microsatellite loci. Kilifi was the most diverse parasite population, followed by Bakoumba and Pikine. n = number of samples, SE = standard error. (DOC) [file pone.0016629.s007.doc]

**Table S3**

| **Parasite population** | ***n*** | **Alleles per locus** | | | **Heterozygosity** | | |
| --- | --- | --- | --- | --- | --- | --- | --- |
|  |  | ***Mean*** | **SE** | **Range** | ***Mean*** | ***SE*** | ***Range*** |
| **Kilifi** | 47 | 9.1 | 1 | 4-16 | 0.79 | 0.03 | 0.55-0.92 |
| **Pikine** | 30 | 6.8 | 0.5 | 4-10 | 0.71 | 0.06 | 0.35-0.96 |
| **Bakoumba** | 24 | 7.5 | 0.9 | 3-14 | 0.76 | 0.06 | 0.27-0.95 |
