# Supplementary material for: A Molecular Epidemiological Study of var Gene Diversity to Characterize the Reservoir of Plasmodium falciparum in Humans in Africa
Source: PLoS One. 2011 Feb 9;6(2):e16629. doi: 10.1371/journal.pone.0016629 (PMC3036650; doi:10.1371/journal.pone.0016629)
Supplement: Table S4 — Geographic differentiation of African local populations based on analysis of 12 microsatellite loci. Wrights FST values for population comparisons by microsatellite alleles calculated using FSTAT [6]. (DOC) [file pone.0016629.s008.doc]

**Table S4**

|  | **Kilifi** | **Pikine** |
| --- | --- | --- |
| **Pikine** | 0.039* |  |
| **Bakoumba** | 0.019 | 0.019 |
| **Kilifi + Bakoumba** |  | 0.033* |

**P* <0.01
