# Supplementary material for: A Molecular Epidemiological Study of var Gene Diversity to Characterize the Reservoir of Plasmodium falciparum in Humans in Africa
Source: PLoS One. 2011 Feb 9;6(2):e16629. doi: 10.1371/journal.pone.0016629 (PMC3036650; doi:10.1371/journal.pone.0016629)
Supplement: Table S5 — African var types shared across populations. From the datasets analyzed, this table lists the African var types also found in Amele and/or Porto Velho, as well as var types found in all three African populations. Under ‘Accession Number,’ a representative African sequence is listed. The table indicates the populations in which each type was found. Two shared var types, DQ135232 and DQ135587, demonstrated high homology to var1CSA, an unusual semi-conserved var gene [7]. No common sequences matched the conserved var gene var2csa, however the primers were not designed to amplify this unique gene. (DOC) [file pone.0016629.s009.doc]

**Table S5**

|  | **Presence in Local Population Samples** | | | | |  |
| --- | --- | --- | --- | --- | --- | --- |
|  | **Asia** | **S. America** | **Africa** | | |  |
| **Accession Number** | **Amele, PNG** | **Porto Velho, Brazil** | **Bakoumba, Gabon** | **Pikine, Senegal** | **Kilifi, Kenya** | **Comments** |
| DQ134449 | X | X | X | X | X |  |
| DQ135349 | X | X | X | X | X |  |
| DQ135232 | X | X | X |  | X | *var1csa* |
| DQ134089 | X | X | X |  |  |  |
| DQ135268 | X | X | X |  |  |  |
| DQ134580 | X |  | X | X |  |  |
| DQ134514 | X |  | X |  |  |  |
| DQ134044 | X |  | X |  |  |  |
| DQ134281 | X |  | X |  |  |  |
| DQ134388 | X |  | X |  |  |  |
| DQ134264 | X |  | X |  |  |  |
| DQ135441 | X |  | X |  |  |  |
| DQ135208 | X |  | X |  |  |  |
| DQ135346 | X |  | X |  |  |  |
| DQ135354 | X |  | X |  |  |  |
| DQ135382 | X |  | X |  |  |  |
| DQ135248 | X |  | X |  |  |  |
| DQ135181 | X |  | X |  |  |  |
| HQ733219 | X |  |  | X |  |  |
| HQ732800 | X |  |  |  | X |  |
| HQ733090 | X |  |  |  | X |  |
| HQ732307 | X |  |  |  | X |  |
| DQ135587 |  | X | X | X | X | *var1csa* |
| DQ134912 |  | X | X | X |  |  |
| DQ135480 |  | X | X |  | X |  |
| DQ135201 |  | X | X |  |  |  |
| DQ135481 |  | X | X |  |  |  |
| DQ134565 |  | X | X |  |  |  |
| HQ733339 |  | X |  | X |  |  |
| HQ733483 |  | X |  | X |  |  |
| DQ135244 |  |  | X | X | X |  |
| DQ135261 |  |  | X | X | X |  |
| DQ135559 |  |  | X | X | X |  |
| DQ135246 |  |  | X | X | X |  |
| DQ135224 |  |  | X | X | X |  |
| DQ134926 |  |  | X | X | X |  |
| DQ134681 |  |  | X | X | X |  |
