# Supplementary material for: A Molecular Epidemiological Study of var Gene Diversity to Characterize the Reservoir of Plasmodium falciparum in Humans in Africa
Source: PLoS One. 2011 Feb 9;6(2):e16629. doi: 10.1371/journal.pone.0016629 (PMC3036650; doi:10.1371/journal.pone.0016629)
Supplement: Table S6 — Chi-square test on the distribution var sequence frequencies among the five populations. Differences in frequency distribution among the five populations are statistically significant by χ2 analysis (χ2 = 1152; p<0.0001). (DOC) [file pone.0016629.s010.doc]

**Table S6**

|  | **Population sample** | | | | |
| --- | --- | --- | --- | --- | --- |
|  | **Bakoumba** | **Pikine** | **Kilifi** | **Amele** | **Porto Velho** |
| **Number of *var* sequences found in only one isolate** | 597 | 554 | 622 | 103 | 59 |
| **Number of *var* sequences found in two or more isolates** | 190 | 118 | 77 | 347 | 384 |
