# Supplementary material for: A Molecular Epidemiological Study of var Gene Diversity to Characterize the Reservoir of Plasmodium falciparum in Humans in Africa
Source: PLoS One. 2011 Feb 9;6(2):e16629. doi: 10.1371/journal.pone.0016629 (PMC3036650; doi:10.1371/journal.pone.0016629)
Supplement: Text S1 — Supporting references. (DOC) [file pone.0016629.s011.doc]

**Text S1: Supporting references:**

1. Shen T-J, Chao A, Lin C-F (2003) Predicting the number of new species in further taxonomic sampling. Ecology 84: 798-804.

2. Colwell RK, Coddington JA (1994) Estimating Terrestrial Biodiversity through Extrapolation. Philosophical Transactions of the Royal Society B: Biological Sciences 345: 101-118.

3. Barry AE, Leliwa-Sytek A, Tavul L, Imrie H, Migot-Nabias F, et al. (2007) Population Genomics of the Immune Evasion (var) Genes of Plasmodium falciparum. PLoS Pathog 3: e34.

4. Albrecht L, Castiñeiras C, Carvalho BO, Ladeia-Andrade S, Santos da Silva N, et al. (2010) The South American Plasmodium falciparum var gene repertoire is limited, highly shared and possibly lacks several antigenic types. Gene 453: 37-44.

5. Albrecht L, Merino EF, Hoffmann EHE, Ferreira MU, de Mattos Ferreira RG, et al. (2006) Extense variant gene family repertoire overlap in Western Amazon Plasmodium falciparum isolates. Molecular and Biochemical Parasitology 150: 157-165.

6. Goudet J (1995) FSTAT (Version 1.2): A Computer Program to Calculate F-Statistics. J Hered 86: 485-486.

7. Kyes SA, Kraemer SM, Smith JD (2007) Antigenic Variation in *Plasmodium* *falciparum*: Gene Organization and Regulation of the *var* Multigene Family. Eukaryotic Cell 6: 1511-1520.
